# Factors Influencing the Emergence and Spread of HIV Drug Resistance Arising from Rollout of Antiretroviral Pre-Exposure Prophylaxis (PrEP)

**DOI:** 10.1371/journal.pone.0018165

**Published:** 2011-04-15

**Authors:** Ume L. Abbas, Gregory Hood, Arthur W. Wetzel, John W. Mellors

**Affiliations:** 1 Department of Infectious Diseases, Cleveland Clinic Foundation, Cleveland, Ohio, United States of America; 2 Pittsburgh Supercomputing Center, Pittsburgh, Pennsylvania, United States of America; 3 Division of Infectious Diseases, School of Medicine, University of Pittsburgh, Pittsburgh, Pennsylvania, United States of America; University of Cape Town, South Africa

## Abstract

**Background:**

The potential for emergence and spread of HIV drug resistance from rollout of antiretroviral (ARV) pre-exposure prophylaxis (PrEP) is an important public health concern. We investigated determinants of HIV drug resistance prevalence after PrEP implementation through mathematical modeling.

**Methodology:**

A model incorporating heterogeneity in age, gender, sexual activity, HIV infection status, stage of disease, PrEP coverage/discontinuation, and HIV drug susceptibility, was designed to simulate the impact of PrEP on HIV prevention and drug resistance in a sub-Saharan epidemic.

**Principal Findings:**

Analyses suggest that the prevalence of HIV drug resistance is influenced most by the extent and duration of inadvertent PrEP use in individuals already infected with HIV. Other key factors affecting drug resistance prevalence include the persistence time of transmitted resistance and the duration of inadvertent PrEP use in individuals who become infected on PrEP. From uncertainty analysis, the median overall prevalence of drug resistance at 10 years was predicted to be 9.2% (interquartile range 6.9%–12.2%). An optimistic scenario of 75% PrEP efficacy, 60% coverage of the susceptible population, and 5% inadvertent PrEP use predicts a rise in HIV drug resistance prevalence to only 2.5% after 10 years. By contrast, in a pessimistic scenario of 25% PrEP efficacy, 15% population coverage, and 25% inadvertent PrEP use, resistance prevalence increased to over 40%.

**Conclusions:**

Inadvertent PrEP use in previously-infected individuals is the major determinant of HIV drug resistance prevalence arising from PrEP. Both the rate and duration of inadvertent PrEP use are key factors. PrEP rollout programs should include routine monitoring of HIV infection status to limit the spread of drug resistance.

## Introduction

Antiretroviral (ARV) pre-exposure prophylaxis (PrEP) is a promising HIV prevention strategy [1], [2]. There is widespread concern, however, about the potential emergence and spread of HIV drug resistance arising from PrEP rollout, particularly in resource-constrained settings, where antiretroviral treatment options are limited. This concern is amplified by the possibility that the same antiretroviral drugs will be used for both treatment and PrEP. Insight is needed into factors influencing the emergence and spread of HIV drug resistance at the population level from PrEP [3]. We therefore used a mathematical model to analyze the potential impact of orally administered PrEP on HIV drug resistance outcomes through simulation of different PrEP implementation scenarios. The focus of the current work was to identify major determinants of HIV drug resistance prevalence after PrEP implementation rather than prediction of actual outcomes.

## Methods

### Model Structure

We have developed and analyzed a population model of heterosexual HIV transmission and disease progression to assess the impact of PrEP implementation [4]. In brief, the model consists of coupled, nonlinear differential equations describing population and epidemiological stratifications based on gender, age, sexual activity, PrEP use status (on/off), infection status (susceptible/infected), stage of HIV infection, and HIV drug susceptibility. Model input parameters were chosen to simulate a mature epidemic in southern sub-Saharan Africa [4]. Parameter assignments were made from recent literature on HIV disease progression, infectivity, sexual behavior and the emergence, transmission and persistence of HIV drug resistance.

**Figure 1:**
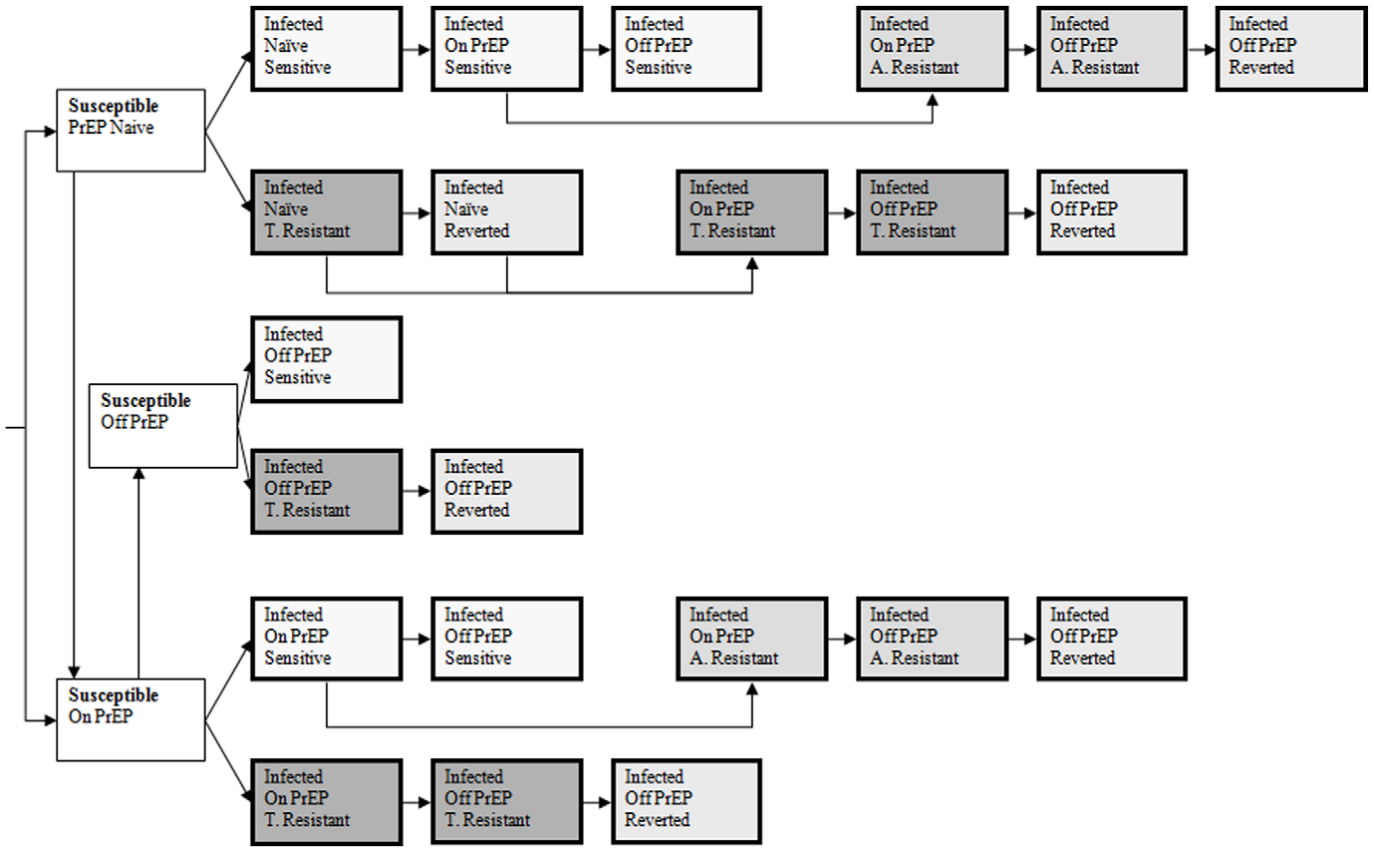
Simplified Model Flow Diagram. **A. Resistant = acquired resistance and T.** Resistance = transmitted resistance.

For the present work, we extended our published model [4] by incorporating detailed representation of HIV drug resistance, both transmitted and acquired, arising from PrEP as outlined in Figure 1, and with parameter assignments listed in Table 1. Model equations and details are provided in Appendix S1. In addition to PrEP use in susceptible individuals, we model inadvertent PrEP use in individuals previously HIV-infected (pre-infected) as well as those who become infected while on PrEP (post-infected). The final model describes a sexually active population (15–49 year-olds) that is stratified into many different states based on epidemiologic, demographic and behavioral characteristics, including 22 unique HIV drug susceptibility strata described below.

**Table 1:** Model Parameters for PrEP Scenarios.

PARAMETER	UNIT	SENSITIVITY	SCENARIO			REFERENCE
		*LHS* † * RANGE*	*OPTIMISTIC*	*REALISTIC*	*PESSIMISTIC*	
Fraction of individuals enrolled into PrEP (coverage)	per year	0.15–0.60	0.60	0.30	0.15	Assumption
Time period to achieve target coverage	year	1–10	1	5	10	Assumption
Efficacy of PrEP against sensitive virus (ξ)	-	0.25–0.75	0.75	0.50	0.25	Assumption
(Relative) Efficacy of PrEP against resistant virus (ξ^R^ = ι*ξ)	-	0.00–0.25 * ξ	0.25* ξ	0.125* ξ	0* ξ	Assumption
Adherence (θ)	-	0.25–0.75	0.75	0.50	0.25	Assumption
PrEP discontinuation rate in susceptible individuals	per year	0.05–0.25	0.05	0.10	0.25	Assumption
Duration of inadvertent PrEP use in those who become infected on PrEP	year	0.5–3	0.5	1	3	Assumption
Rate of inadvertent PrEP uptake in previously-infected individuals	per year	0.05–0.25	0.05	0.10	0.25	Assumption
Duration of inadvertent PrEP use in previously-infected individuals	year	0.5–3	0.5	1	3	Assumption
Time to development of acquired resistance in inadvertent PrEP users who become infected on PrEP (t_1_)	year	0.167–0.5	0.5	0.25	0.167	[5]
Rate of development of acquired resistance in inadvertent PrEP users who become infected on PrEP	per year	derived	−LN(1−0.99*θ)/t_1_	−LN(1−0.99*θ)/t_1_	−LN(1−0.99*θ)/t_1_	[46]
Time to development of acquired resistance in inadvertent PrEP users who are previously infected (t_2_)	year	0.083–0.25	0.25	0.125	0.083	[38], [39]
Rate of development of acquired resistance in inadvertent PrEP users who are previously infected	per year	derived	−LN(1−0.99*θ)/t_2_	−LN(1−0.99*θ)/t_2_	−LN(1−0.99*θ)/t_2_	[46]
Infectivity of donor with transmitted resistance*	per act	0.5–1.0 * γ^Ω^	0.5	0.75	1	[7], [9], [14], [15], [16]
Infectivity of donor with acquired resistance*	per act	0.5–1.0 * γ^Ω^	0.5	0.75	1	[7], [9], [14]
Probability of transmission of resistant versus sensitive virus from donor with transmitted resistance	-	0.75–1.0	0.75	0.9	1	[17], [18], [19]
Probability of transmission of resistant versus sensitive virus from a donor with acquired resistance	-	0.5–1.0	0.5	0.75	1	[17], [18], [19]
Probability of transmission of resistant versus sensitive virus from a donor with wild-type or reverted to wild-type virus to a recipient on PrEP	-	0.01–0.25	0.01	0.05	0.25	Assumption
Persistence time of transmitted resistance in recipients not on PrEP	year	1–5	1	2	5	[8], [10], [22]
Persistence time of transmitted resistance in recipients after PrEP discontinuation	year	1–5	1	2	5	[8], [10], [22]
Persistence time of acquired resistance after PrEP discontinuation	year	0.083–1	0.083	0.5	1	[7], [9], [11]
Factor increase in rates of sexual partnership change of individuals, both susceptible and infected, while on PrEP (i.e., risk compensation)	-	1.0–2.0	1.0–2.0	1.0–2.0	1.0–2.0	Assumption

HIV disease progression [4] was assumed the same for drug-resistant and drug-sensitive virus because: i) a temporary predominance of drug-resistant mutants was assumed in the model; and ii) though lower viremia has been observed in the experimental setting [14], it is unknown whether PrEP would attenuate the course of HIV infection.

HIV infectivity and disease progression [4] in individuals with drug-sensitive virus were assumed to be unchanged by their PrEP status.

*Relative to infectivity (per sex act probability of transmission) of donor with wild-type virus based on stage of infection, γ^Ω^ [4].

† Latin Hypercube Sampling (uniform distribution).

#### Effectiveness of PrEP

Our model represents the transmission of HIV as a Poisson process [3]. The probability of transmission per heterosexual partnership, *β*, between an individual (on PrEP) of gender *g*, activity level *k*, and age *i*, with an (infected) individual of opposite gender *g′*, activity level *l* and age *j* is given by:
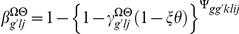
where *Ψ* is the number of sex acts within the partnership; *γ* is the probability of HIV transmission per sex-act (infectivity) based on the disease stage, *Ω*, and drug resistance status, *Θ*, of the infected partner; and *ξθ* is the effectiveness of PrEP. Effectiveness is defined as the probability of preventing HIV transmission per sex-act through PrEP and is given by the product of the average efficacy of PrEP, *ξ* (the degree of protection provided, from HIV transmission per sex-act) and the average level of adherence to PrEP, *θ* (assuming once daily dosing of a single antiretroviral drug and that doses are missed at random). In a partnership, where the infected partner harbors major drug-resistant variants (discussed below), the probability of transmission of resistant virus is *υβ*, while that of wild-type virus is *(1−υ)β*, and the effectiveness of PrEP against resistant virus is *ιξθ*. The parameters *ξ*, *θ*, *υ* and *ι* assume values between 0 and 1 (Table 1).

#### Modeling Drug Resistance

We sub-classified HIV-infected individuals based on their PrEP status (naïve, on PrEP or off PrEP), HIV drug susceptibility (drug-sensitive or drug-resistant), type of drug resistance (transmitted or acquired), and virus population dynamics of drug-resistant HIV (persistence of resistance or reversion of resistance, the latter either from genetic reversion of virus to wild-type or overgrowth of resistant virus by wild-type virus) into 22 different HIV drug susceptibility strata (Figure 1 and Tables 1, 2). Our key model assumptions for HIV drug resistance are as follows. In an HIV-infected individual, the virus population is comprised of a set of related variants, termed a quasispecies [5]. Before the introduction of PrEP in antiretroviral naïve persons, the major (predominant) variants are wild-type and drug-sensitive. After the introduction of PrEP, drug-sensitive virus or drug-resistant variants may predominate. Individuals with predominantly drug-sensitive or drug-resistant variants may probabilistically transmit either drug-sensitive or drug-resistant virus to their sexual partners (Table 1). *Transmitted resistance* (Table 2) may occur from: i) a donor having a majority population of drug-resistant variant to a recipient either receiving or not receiving PrEP; or ii) a donor having a majority population of drug-sensitive virus to a recipient receiving PrEP. *Acquired resistance* may occur from the selection of drug-resistant virus in individuals with drug-sensitive virus, who were either previously infected or became infected while receiving PrEP [5], [6]. Upon removal of drug selection, either by discontinuation of PrEP [7] or transmission to an individual not on PrEP (never started or discontinued) [8], the drug-resistant virus reverts to drug-sensitive virus after a period of persistence, either from overgrowth of resistant variants by wild-type variants or genetic reversion of the resistant variants to wild-type [9], [10], [11]. Prior to reversion, drug-resistant variants comprise the majority population, whereas following reversion they become a minor population [6], [12], [13]. Compared to individuals with wild-type virus, individuals with majority resistant variants may have: i) decreased per act probability of transmission of HIV to their sexual partners (infectivity) because of reduced transmission fitness or from lower virus levels, the latter either from continued antiretroviral activity of PrEP [7], [14] or from reduced viral replicative fitness [9], [15], [16]; and ii) increased probability of sexual transmission of drug-resistant strains versus drug-sensitive strains [17], [18], [19]. Individuals with minority drug-resistant variants are similar to individuals with majority wild-type variants in terms of HIV transmission and disease progression. The re-emergence of drug-resistant variants from antiretroviral therapy was not modeled [20], [21], [22].

**Table 2:** Model Cases for HIV Transmission.

	HIV Donor		HIV Recipient	
Case	PrEP Status	Majority Variant	PrEP Status	Transmitted Variant
1	−	Wild-type	−	Sensitive
2	+	Wild-type	−	Sensitive
3	−	Wild-type	+	Sensitive
4	−	Wild-type	+	Resistant
5	+	Wild-type	+	Sensitive
6	+	Wild-type	+	Resistant
7	−	Acquired Resistant	−	Sensitive
8	−	Acquired Resistant	−	Resistant
9	−	Acquired Resistant	+	Sensitive
10	−	Acquired Resistant	+	Resistant
11	+	Acquired Resistant	−	Sensitive
12	+	Acquired Resistant	−	Resistant
13	+	Acquired Resistant	+	Sensitive
14	+	Acquired Resistant	+	Resistant
15	−	Transmitted Resistant	−	Sensitive
16	−	Transmitted Resistant	−	Resistant
17	−	Transmitted Resistant	+	Sensitive
18	−	Transmitted Resistant	+	Resistant
19	+	Transmitted Resistant	−	Sensitive
20	+	Transmitted Resistant	−	Resistant
21	+	Transmitted Resistant	+	Sensitive
22	+	Transmitted Resistant	+	Resistant
23	−	Reverted to Wild-type	−	Sensitive
24	−	Reverted to Wild-type	+	Sensitive
25	−	Reverted to Wild-type	+	Resistant

#### Model Output and Introduction of PrEP

The model's dynamical behavior was investigated using numerical methods. The key model outputs were: i) HIV incidence; ii) HIV prevalence; iii) cumulative new HIV infections; iv) proportion of cumulative new infections with transmitted resistance; v) overall prevalence of HIV drug resistance (transmitted plus acquired); vi) prevalence of transmitted resistance; and vii) prevalence of acquired resistance. PrEP was introduced (once daily oral dosing of a single antiretroviral drug, e.g. tenofovir disoproxil fumarate) at endemic equilibrium when HIV prevalence in sexually active adults (15–49 year-olds) was approximately 20%. We made comparisons between the epidemics with and without PrEP at each simulation time-step over a 10 year interval after PrEP introduction.

### Sensitivity Analyses

We performed sensitivity analyses [23], [24] to determine the relative influence of PrEP-related input parameters (Table 1) on outcomes and examined our prediction uncertainty [25]. Univariate sensitivity analyses were performed using batch simulations in which the PrEP-related input parameters were individually varied, over their entire range, followed by examination of tornado and contour plots of output. For multivariate time-dependent sensitivity analyses, we performed two sets of 10,000 runs using Latin hypercube sampling to simulate HIV epidemics and the implementation of PrEP, either with or without risk compensation (increase in rate of sex partner change) occurring in the population on PrEP. We rank transformed input and output data obtained from simulations and derived standardized rank regression coefficients (SRRCs) [26]. The strength and nature of the relationship between an input parameter and the outcome are given by the size and sign (+/−) of the relevant SRRC. Because we sampled the input parameters independently, the fraction of variance in model outcome explained by each parameter is given by the square of its SRRC [27], [28]. In addition to the model's sensitivity to parameter uncertainty, we studied the model's sensitivity to key assumptions by comparing the outputs of an original model with those obtained using different structural assumptions (singly or combined) including no PrEP use in previously-infected individuals.

### PrEP Scenarios

The impact of PrEP was next determined by simulating three different scenarios: optimistic, realistic and pessimistic (Table 1). For each of these scenarios, we simulated PrEP implementation with proportional PrEP coverage in the following susceptible populations: i) the sexually active population in general (non-targeted strategy); ii) targeted to the group 15–20 years of age (targeted-by-age strategy); iii) targeted to the female population (targeted-by-gender strategy); and iv) targeted to the two highest sexual activity levels (targeted-by-activity strategy). In addition, the scenarios (optimistic, realistic and pessimistic) represented inadvertent PrEP use in the previously-infected population (rates/year of 5%, 10% and 25%) as well as in all the individuals infected on PrEP, for a variable period of time.

## Results

Our mathematical model stratifies the study population by gender, age, sexual activity level, PrEP use, HIV infection status, disease stage and HIV drug susceptibility (Figure 1), and its dynamical behavior is analyzed numerically. We introduced PrEP at endemic equilibrium and simulated optimistic, realistic and pessimistic scenarios (Table 1). For each scenario we simulated four strategies of PrEP implementation: i) in the sexually active population in general (non-targeted strategy); ii) targeted to the group 15–20 years of age (targeted-by-age strategy); iii) targeted to the female population (targeted-by-gender strategy); and iv) targeted to the two highest sexual activity levels (targeted-by-activity strategy). To determine the epidemiological impact of PrEP, we compared epidemics with and without PrEP for up to 10 years for: i) HIV incidence; ii) HIV prevalence; iii) cumulative new HIV infections; in addition we determined outcomes of drug resistance from PrEP including iv) proportion of cumulative new infections with transmitted resistance; v) overall prevalence of HIV drug resistance (transmitted plus acquired); vi) prevalence of transmitted resistance; and vii) prevalence of acquired resistance.

### Factors Influencing Impact of PrEP on Transmission vs. HIV Drug Resistance

Table 3 shows multivariate sensitivity analyses of model outcomes after 10 years of PrEP implementation in the absence of risk compensation. The key parameters influencing the impact of PrEP on HIV prevention were different from those affecting the prevalence of HIV drug resistance. Specifically, the extent of PrEP coverage (SRRC = 0.52) explained 26.9% of the variance in cumulative infections prevented. The level of PrEP adherence (SRRC = 0.49), PrEP efficacy against wild-type virus (SRRC = 0.42), infectivity of individuals with acquired resistance (SRRC = −0.32), and the rate of PrEP discontinuation in susceptible individuals (SRRC = −0.23) explained 24%, 17.5%, 9.9% and 5.4% of the variance in infections prevented, respectively.

**Table 3:** Sensitivity Analysis of Outcomes after 10 years of PrEP Implementation.

Model Input*	Model Output			
	Cumulative New Infections Prevented	Prevalence of Overall Resistance†	Prevalence of Transmitted Resistance†	Prevalence of Acquired Resistance†
	***Standardized Rank Regression Coefficients (% variance explained*** § ***)***			
PrEP Coverage	0.52 (26.9)			
Adherence	0.49 (24.0)			
Efficacy of PrEP against sensitive virus	0.42 (17.5)			
Infectivity of individuals with acquired resistance	−0.32 (9.9)			
PrEP discontinuation rate in susceptible individuals	−0.23 (5.4)			
Duration of inadvertent PrEP use in pre-infected individuals		0.62 (38.8)	0.32 (10.2)	0.74 (54.1)
Rate of inadvertent PrEP uptake in pre-infected individuals		0.34 (11.7)	0.32 (10.5)	0.27 (7.5)
Duration of inadvertent PrEP use in post-infected individuals		0.30 (9.2)		0.32 (10.0)
Persistence time of transmitted resistance		0.28 (7.6)	0.53 (28.0)	
Persistence time of acquired resistance				0.25 (6.0)

*Parameters that contribute 5% or more of the variance in the model outcome are shown (SRRC^2^≥0.05). The reported coefficients were significant with a p-value≤0.05.

§ Of the total variance in the predicted outcome explained by the regression model. The respective R^2^ values were: 0.91 (cumulative infections prevented); 0.85 (overall prevalence of resistance); 0.89 (prevalence of transmitted resistance); 0.85 (prevalence of acquired resistance); and 0.89 (resistant cumulative infections).

† Proportion of cases with drug-resistant infection in the infected population.

By contrast, the overall prevalence of drug resistance was influenced most by the duration of inadvertent PrEP use (SRRC = 0.62) and the rate of PrEP uptake (SRRC = 0.34) in previously-infected individuals. Together these two parameters explained 50.5% of the variance in overall prevalence of resistance after 10 years. Not surprisingly, the prevalence of transmitted resistance after 10 years was most influenced by the persistence time of transmitted resistance (SRRC = 0.53), explaining 28% of the variance. The rate of PrEP uptake and duration of inadvertent use in previously-infected individuals (SRRC = 0.32) explained another 10.5% and 10.2% of variance in transmitted resistance, respectively. The prevalence of acquired resistance was most sensitive to the duration of inadvertent PrEP use (SRRC = 0.74) and its rate of uptake (SRRC = 0.27) in previously-infected individuals; together these parameters explained 61.6% of the variance in the prevalence of acquired resistance after 10 years. Likewise, the rate (SRRC = 0.40) and duration (SRRC = 0.36) of inadvertent PrEP use in previously-infected individuals were most influential for the proportion of cumulative new infections with transmitted resistance, explaining 28.8% of the variance in this outcome (data not shown). Factors influencing the prevalence of drug resistance when risk compensation was assumed were similar to the above (data not shown).

### Scenario Analysis

**Figure 2:**
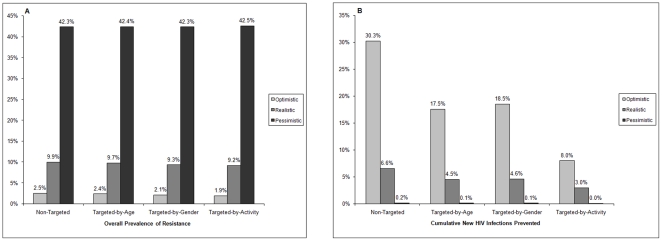
Outcomes after10 years of PrEP rollout in optimistic, realistic and pessimistic scenarios with four different strategies. Panel A shows overall prevalence of HIV drug resistance and Panel B shows cumulative new HIV infections prevented.

**Figure 3:**
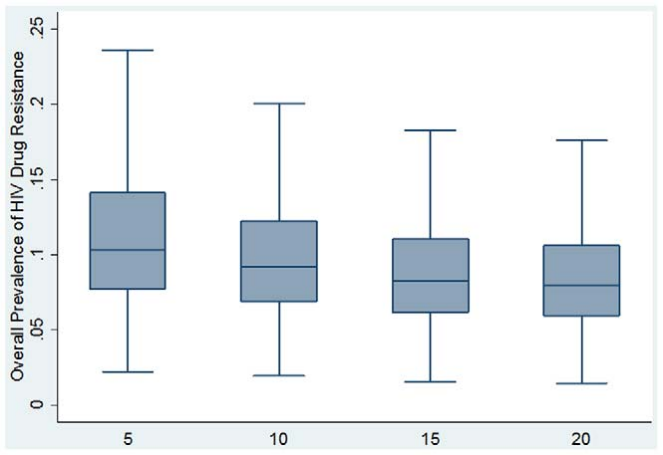
Overall prevalence of HIV drug resistance after 5, 10, 15 and 20 years of PrEP rollout predicted by uncertainty analysis. For each time point, results of 10,000 simulations are shown as a box-and-whisker plot; representing the median, upper and lower quartiles, and maximum and minimum values.

Table 1 shows the PrEP-related input parameters for the three different scenarios. Table 4 compares the epidemiologic outcomes in optimistic, realistic and pessimistic scenarios 10 years after the introduction of PrEP. The overall prevalence of drug resistance was highest for the pessimistic scenario (42.3%), but was minimal for the optimistic scenario (2.5%), illustrating the importance of the key scenario parameters on resistance prevalence (Figure 2A). With uncertainty analysis (Figure 3), the median overall prevalence of drug resistance at 10 years was 9.2% (interquartile range 6.9%–12.2%), similar to the resistance prevalence for the realistic scenario (9.9%). For both the optimistic and the realistic scenario, the non-targeted strategy generated the most resistance, whereas the targeted-by-activity strategy generally produced the least resistance with the following rank order of resistance prevalence: non-targeted>targeted-by-age>targeted-by-gender>targeted-by-activity. By contrast, high resistance prevalence was seen with the pessimistic scenario across the four different strategies (Table 4 and Figure 2A).

**Table 4:** Outcomes for Optimistic (O), Realistic (R) and Pessimistic (P) Scenarios after 10 Years of PrEP Implementation.

	Non-Targeted			Targeted-by-Age			Targeted-by-Gender			Targeted-by-Activity		
	O	R	P	O	R	P	O	R	P	O	R	P
Overall prevalence* of resistance	2.5%	9.9%	42.3%	2.4%	9.7%	42.4%	2.1%	9.3%	42.3%	1.9%	9.2%	42.5%
Prevalence* of transmitted resistance	0.4%	2.9%	27.1%	0.3%	2.7%	27.0%	0.2%	2.5%	26.9%	0.2%	2.5%	26.9%
Prevalence* of acquired resistance	2.2%	7.0%	15.2%	2.1%	7.0%	15.4%	1.9%	6.8%	15.5%	1.7%	6.6%	15.6%
Cumulative new infections prevented	30.3%	6.6%	0.2%	17.5%	4.5%	0.1%	18.5%	4.6%	0.1%	8.0%	3.0%	0.0%
Resistant cumulative infections†	2.2%	8.3%	40.3%	1.5%	7.4%	39.9%	1.3%	7.0%	39.7%	1.3%	7.1%	39.7%
Decline in HIV prevalence	26.2%	6.0%	0.2%	16.6%	4.2%	0.1%	16.2%	4.2%	0.1%	7.1%	2.7%	0.0%
Decline in HIV incidence	32.3%	7.4%	0.2%	25.4%	6.0%	0.1%	20.2%	5.3%	0.1%	8.6%	3.2%	0.0%

*Proportion of cases with drug-resistant infection in the infected population.

† Proportion of cumulative new infections with transmitted resistance.

For each scenario, the largest decrease in infections was achieved with the non-targeted strategy and the smallest decrease with the targeted-by-activity strategy (Table 4 and Figure 2B). Specifically, a 30.3% reduction in infections occurred for the optimistic scenario, 6.6% for the realistic scenario and 0.2% for the pessimistic scenario with the non-targeted strategy. These reductions fell to 8%, 3% and 0%, respectively, with the targeted-by-activity strategy. However, the proportion of cumulative infections with transmitted resistance also fell with the targeted-by-activity strategy: from 2.2% to 1.3% for the optimistic scenario: from 8.3% to 7.1% for the realistic scenario; and minimally from 40.3% to 39.7% for the pessimistic scenario. The targeted-by age and targeted by-gender strategies yielded intermediate declines in infections (17.5%, 4.5%, 0.1% and 18.5%, 4.6%, 0.1%, respectively). Overall, the declines in HIV prevalence and incidence were highest for the optimistic scenario (26.2% and 32.3%, respectively, for the non-targeted strategy) with minimal changes observed with the pessimistic scenario (0.2% for the non-targeted strategy).

**Figure 4:**
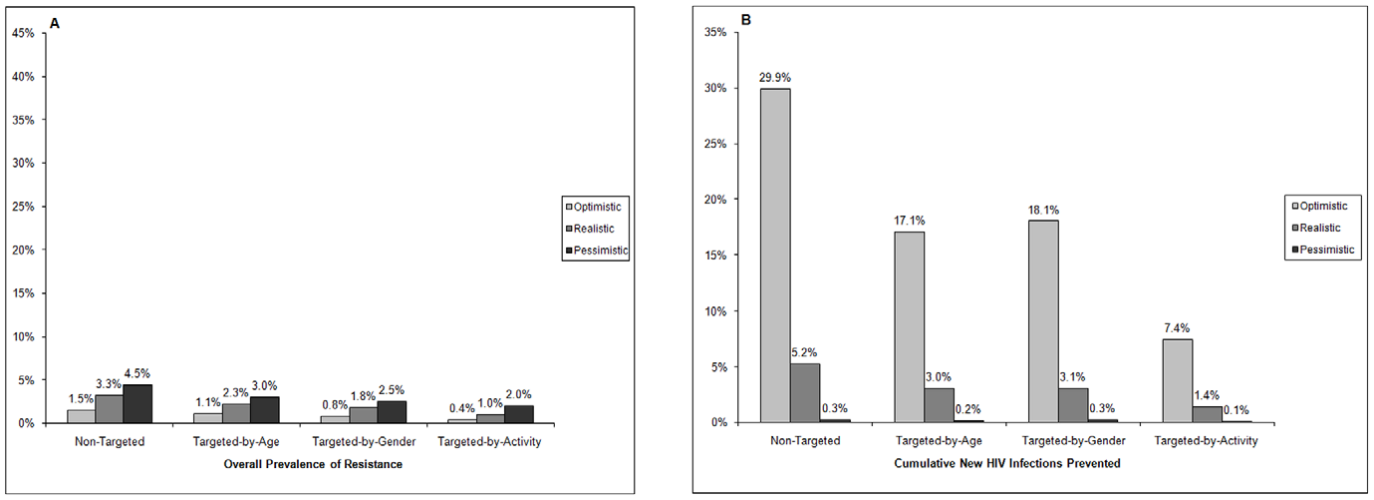
Outcomes after10 years of PrEP rollout assuming no inadvertent PrEP uptake in previously infected individuals for optimistic, realistic and pessimistic scenarios, with four different strategies. Panel A shows overall prevalence of HIV drug resistance and Panel B shows cumulative new HIV infections prevented.

Univariate sensitivity analyses of resistance prevalence confirmed that the most important factors affecting resistance prevalence were the rate and duration of use of inadvertent PrEP in previously-infected individuals. When no inadvertent PrEP use in previously infected individuals was assumed, there was a major decline in the prevalence of drug resistance (Figure 4A), particularly in the pessimistic scenario, but only modest changes occurred in infections prevented (Figure 4B). Specifically, the prevalence of overall resistance fell from 2.5% to 1.5% in the optimistic, 9.9% to 3.3% in the realistic and 42.3% to 4.5% in the pessimistic scenario (Figure 2A and 4A).

Using the targeted-by-gender strategy (PrEP targeted to female population), more infections were prevented in women compared to men. These findings were generally robust (data not shown) to single and multiple changes in the model's key structural assumptions including those related to balance in the supply and demand of sexual partnerships in the population over time [29] and infectivity of females on PrEP.

#### Trends in Resistance

**Figure 5:**
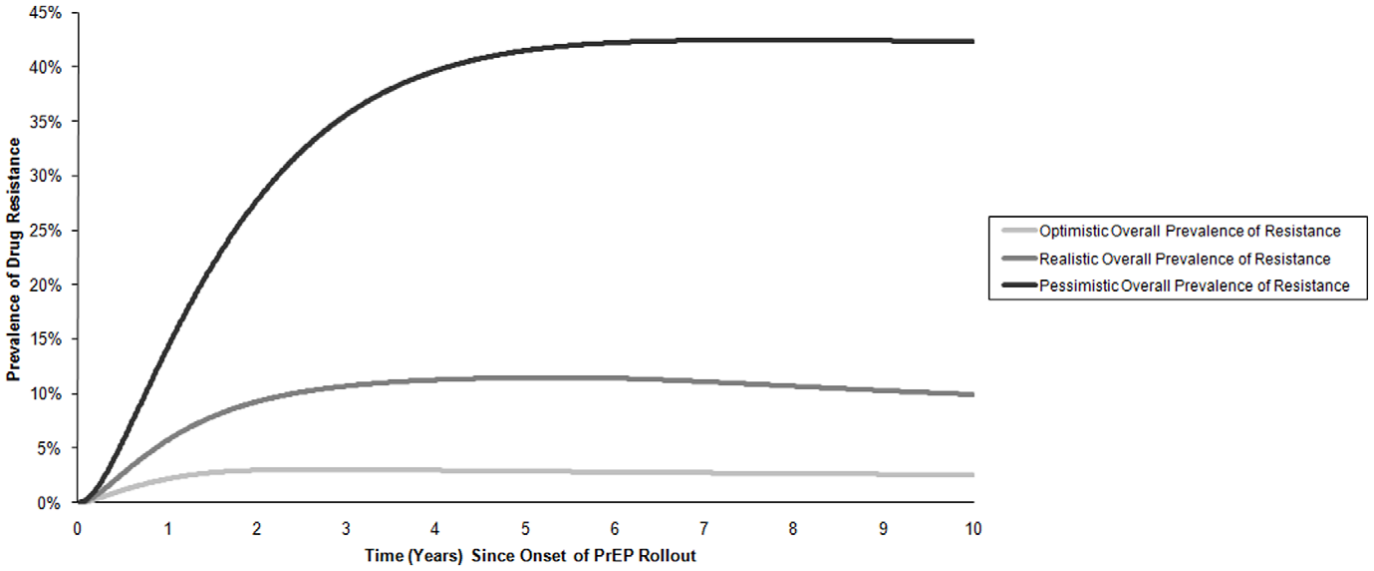
Changes in the prevalence of HIV drug resistance (overall, transmitted, acquired) for10 years after PrEP introduction.

Figure 5 shows the trends in the overall prevalence drug resistance for 10 years after PrEP rollout. After an initial rise, the overall resistance plateaued in the pessimistic scenario, whereas it declined in optimistic and realistic scenarios.

## Discussion

Data from animal studies show that orally administered antiretrovirals can prevent infection of macaques by simian immunodeficiency virus [30]. The safety and efficacy of oral antiretroviral PrEP in humans is being studied in several clinical trials in the United States, Latin America, Africa and Asia [1]; the results of the iPrEx trial are promising [2]. However, these studies are not designed to address the population-level impact of PrEP including potential HIV drug resistance consequences. Uncertainty about HIV drug resistance from PrEP could prevent deployment of PrEP even though it may be shown to prevent HIV infection in clinical trials. Although PrEP implementation has been modeled before by us [4] and others [31], [32], [33], [34], [35], [36], [37], we report here for the first time the main drivers of drug resistance from PrEP in a heterosexual HIV epidemic using a carefully stratified and well-parameterized mathematical model of HIV transmission. Inadvertent PrEP use in already infected individuals is the key driver of increasing drug resistance in a heterosexual population. The prevalence of drug resistance is influenced by both the rate of uptake and duration of use of PrEP in this group. Inadvertent PrEP use in already-infected individuals is not a failure of PrEP per se, but it may occur as an unexpected consequence of PrEP rollout programs and should be assiduously avoided. The duration of PrEP use in susceptible individuals and in individuals infected while on PrEP has less influence on drug resistance outcomes. As expected, the persistence times of transmitted and acquired resistance were critical determinants of the prevalence of transmitted and acquired resistance.

The current model represents a significant refinement of our earlier version in terms of model structure, parameter assignment and scenario design [4]. The current model also includes detailed representations of both transmitted and acquired HIV drug resistance, arising both in individuals who become infected while on PrEP and in previously infected individuals exposed to PrEP. These refinements provided improved precision of model output. Assumptions regarding the effectiveness of PrEP (composite of efficacy and adherence) in our optimistic and neutral scenarios are in general agreement with the results of iPrEx [2], a clinical trial of oral PrEP in men who have sex with men that showed a 44% decrease in HIV incidence (95% confidence interval, 15 to 63).

Notwithstanding model improvements, sensitivity analyses of infections prevented confirm our earlier findings of the impact of PrEP on HIV prevention [4]. The parameters that most influence the impact of PrEP are PrEP coverage, PrEP efficacy and adherence, duration of PrEP use in susceptible individuals, and the infectivity of individuals with acquired resistance. The estimated decreases in HIV infections from PrEP are also in line with our earlier work [4], but are more conservative due to deliberately more pessimistic modeling assumptions, including lower estimates of PrEP efficacy, adherence and coverage, higher rates of PrEP discontinuation in susceptible individuals and significant PrEP exposure in previously-infected individuals.

The results of our scenario analyses provide important insight into potential emergence of HIV drug resistance after PrEP implementation. The non-targeted optimistic and realistic scenarios predicted low to moderate prevalence of drug resistance (2.5% and 9.9% respectively) along with high to moderate decreases in cumulative infections (30.3% and 6.6%, respectively). Uncertainty analysis also predicted moderate levels of overall drug resistance. With targeted optimistic and realistic scenarios, the prevalence of resistance was modestly reduced with considerable erosion (up to 70%) of infections prevented. The prevalence of drug resistance rose to over 40% in the pessimistic scenarios with minimal reduction in HIV infections. Sensitivity analyses showed that the key driver of this negative outcome was the high level of inadvertent PrEP use in the already infected population. When the pessimistic scenarios were re-simulated excluding PrEP use in previously-infected individuals, the prevalence of resistance decreased to 4.5%.

There are some important limitations of our current model structure and the assumptions within it. The precise quantitative detail of our predictions will be affected by variations in the sexual activity patterns of different populations, for which data are very limited, especially on sexual mixing patterns. However, we employed a well-established template of sexual behavior [29], with robust epidemiological and demographic parameterization, broadly applicable to southern sub-Saharan Africa.

The actual impact of PrEP on drug resistance will depend on the PrEP agent or agents used as well as the biological, behavioral and viral characteristics of the HIV-infected population. Although we do not model a specific PrEP agent, we used resistance-related input estimates that would be expected for a single antiretroviral drug used for PrEP such as tenofovir disoproxil fumarate [38], [39]. We did not include combinations of antiretrovirals for PrEP in our initial modeling of drug resistance [1], [40], [41]. Non-human primate studies of PrEP suggest superiority of tenofovir plus emtricitabine over tenofovir alone [42], [43], but it is unknown whether this will be observed in human studies. Natural polymorphisms in HIV subtypes may play an important role in drug resistance, including the propensity of HIV subtype C virus that is predominant in Sub-Saharan Africa for more frequent and rapid development of the K65R tenofovir-resistance mutation noted by some investigators [44], though not by others [45]. To address the substantial uncertainty regarding PrEP-related resistance, we employed wide ranges within plausible bounds for input parameters and performed extensive sensitivity analyses. Our work underscores the need for additional data on the persistence time of transmitted and acquired resistance and the probability of transmission with and without PrEP.

We excluded from our analyses the impact of antiretroviral therapy for infected persons and various other influences on transmission (e.g. STDs, circumcision and condom use). These and other refinements will be addressed in future work. Nevertheless, the important conclusion for our modeling is that the spread of HIV drug resistance could be mitigated by limiting inadvertent PrEP exposure in already infected individuals. To accomplish this, PrEP implementation programs would need to be tightly coupled with HIV testing of individuals who are candidates for PrEP and monitoring of PrEP recipients for HIV infection and drug resistance.

## Supporting Information

Appendix S1 Model Equations and Details.
(DOC)
